# Inflammatory Pseudotumor After Total Hip Arthroplasty‐An Increasingly Recognized Complication

**DOI:** 10.1111/os.70334

**Published:** 2026-06-02

**Authors:** Steve T. L. Pambayi, Zhi Zhao, Hongjun Peng, Ruihao Xia, Xishan Zhu, Wangdui Suolang, Gang Wang, Xiaoou Li, Yi Zeng

**Affiliations:** ^1^ Department of Orthopedics Surgery Orthopedics Research Institute, and National Clinical Research Center for Geriatric, West China Hospital, Sichuan University Chengdu Sichuan China; ^2^ Department of Pulmonary and Critical Care Medicine, Division of Pulmonary Diseases, State Key Laboratory of Biotherapy West China Hospital, West China School of Medicine, Sichuan University Chengdu Sichuan China

**Keywords:** arthroplasty, magnetic resonance imaging, pseudotumor, ultrasonography

## Abstract

Inflammatory pseudotumor (IP) is an increasingly recognized etiological factor in the failure of total hip arthroplasty (THA). It is a non‐neoplastic, non‐infectious mass encircling a hip prosthesis following arthroplasty and may appear as cystic, solid, or mixed tissue. Although initially associated with metal‐on‐metal (MoM) bearing surfaces, IP formation can also occur with other bearing types. Recognized risk factors include female sex, specific genetic predispositions, MoM bearings, large femoral head size, modular femoral stem designs, elevated metal ion levels, and wear debris from polyethylene components. IP contributes to THA failure through both mechanical and inflammatory mechanisms, often leading to pain, instability, or implant loosening. Diagnosis relies heavily on imaging modalities, including plain radiographs, ultrasound, CT, and MRI with metal artifact reduction sequences (MARS‐MRI), which help delineate the extent and characteristics of IP lesions. A multimodal imaging approach is essential for accurate assessment and surgical planning. Management strategies are guided by clinical presentation and radiographic findings, such as IP size and anatomical location, ranging from close observation in asymptomatic cases to revision surgery in symptomatic or progressive disease. IP is therefore an important, imaging‐detectable cause of THA failure, and awareness of its presentation and diagnostic features is critical for timely intervention and improved patient outcomes.

## Graphical abstract

This review systematically elucidates the clinical characteristics and management of inflammatory pseudotumor (IP) across different bearing surfaces, addressing a topic of increasing concern and providing a novel perspective. It synthesizes the five primary risk factors associated with IP, offering clinicians critical reference points.

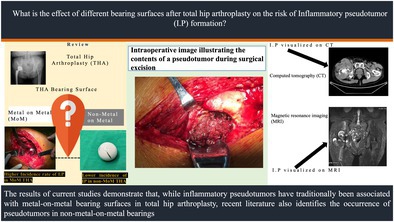

## 1 Introduction

Inflammatory pseudotumor (IP) after total hip arthroplasty (THA) has emerged as a clinically significant yet unsettled complication [1, 2, 3]. This increased recognition may be attributed to advancements in diagnostic imaging, particularly the metal artifact reduction sequence (MARS) magnetic resonance imaging (MRI), and a growing awareness of adverse local tissue reactions (ALTR) [4]. Although much of the literature emphasizes its association with (MoM) metal‐on‐metal bearings, IP arising with a broad range of bearing couples [1, 2, 5] challenges a unitary aetiologic model and invites reappraisal of pathophysiology, risk stratification, and surveillance strategies.

Fundamental questions remain: what are the precise triggers of IP in non‐MoM THA, and how should they be weighted in risk prediction? To what extent do host immunogenetic factors, including HLA profiles, determine susceptibility and course, and can these insights guide prevention or early intervention? How should incidence data be reconciled across bearing types, given heterogeneous diagnostic thresholds and imaging modalities?

## 2 Methods

This review was conducted as a narrative, non‐systematic synthesis of the literature on inflammatory pseudotumor (IP) after total hip arthroplasty (THA), with predefined procedures for literature search and study selection to enhance transparency and reproducibility. A targeted literature search was performed in PubMed, Scopus, and Google Scholar for articles published between January 2010 and July 2025. The core search strategy combined subject terms and keywords as follows: (“inflammatory pseudotumor” OR “pseudotumor” OR “adverse local tissue reaction” OR “ALTR” OR “adverse reaction to metal debris” OR “ARMD”) AND (“total hip arthroplasty” OR “hip replacement” OR “hip resurfacing”). Search strings were adapted for each database according to its specific syntax and filters.

### 2.1 Eligibility Criteria

The PICOS (Population, Intervention, Comparison, Outcome, Study design) framework guided study selection. Population: adults who underwent primary or revision THA or hip resurfacing in whom an inflammatory pseudotumor, ALTR, or ARMD was reported. Intervention/Exposure: presence of a hip prosthesis with exposure to implant wear or corrosion products (including trunnion corrosion), and/or diagnostic or therapeutic interventions related to IP. Comparison: where reported, different bearing surfaces, modular versus non‐modular stems, symptomatic versus asymptomatic patients, or alternative management strategies. Outcomes: incidence or prevalence of IP/ALTR, identified risk factors, diagnostic performance of investigations (laboratory tests, metal ions, imaging), histopathological findings, and clinical or radiographic outcomes of operative or non‐operative management. Study types: peer‐reviewed English‐language human studies, including clinical cohorts, case–control studies, case series, registry analyses, imaging and histopathological studies, and narrative or systematic reviews that provide relevant primary data or synthesize evidence. Studies were excluded if they were non‐hip arthroplasty reports, animal or in vitro experiments without clinical correlation, conference abstracts without full text, or if periprosthetic masses were clearly infectious or malignant rather than inflammatory pseudotumors.

### 2.2 Study Selection and PRISMA‐Style Flow

The database search yielded a total of 241 records across PubMed (*n* = 26), Scopus (*n* = 95), and Google Scholar (*n* = 120). Before screening, 46 records were removed, consisting of 20 duplicates and 26 records identified as non‐relevant formats (e.g., conference abstracts, book chapters). The remaining 195 records were screened by title and abstract, resulting in the exclusion of 53 records that were clearly unrelated (e.g., non‐hip arthroplasty, non‐periprosthetic tumors, animal studies, or purely in vitro work).

**FIGURE 1:**
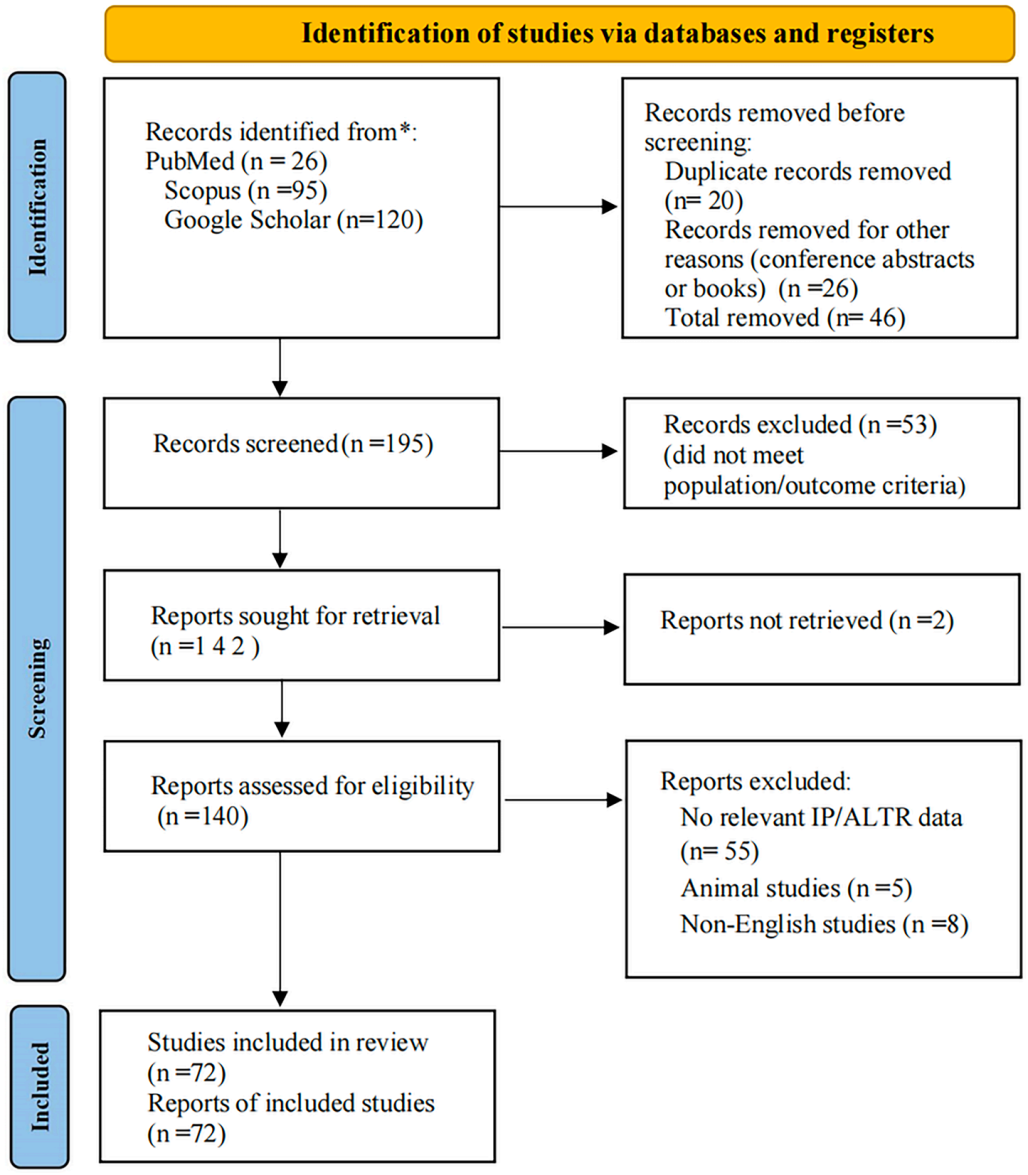
PRISMA‐style flow diagram of study selection for this review of IP after THA, showing records identified across databases, screened, assessed for eligibility, and ultimately included in the qualitative synthesis. Adapted from “Page MJ, McKenzie JE, Bossuyt PM, Boutron I, Hoffmann TC, Mulrow CD, Shamseer L, Tetzlaff JM, Akl EA, Brennan SE, et al. The PRISMA 2020 statement: An updated guideline for reporting systematic reviews. BMJ 2021;372:n71. doi:10.1136/bmj.n71” The PRISMA 2020 statement was used to guide the transparent reporting of the search methodology, even for this narrative review.

Full‐text reports were sought for retrieval for the remaining 142 records; 2 could not be retrieved. The full text of the 140 remaining reports was assessed against the predefined PICOS‐based eligibility criteria. At this stage, 68 reports were excluded: 55 did not contain relevant IP/ALTR outcome data, 5 were animal studies, and 8 were non‐English studies. The final set of studies included in the review (*n* = 72) formed the basis for the qualitative synthesis. A PRISMA‐style flow diagram summarizing this process is presented in Figure 1.

### 2.3 Data Extraction and Synthesis

For each included study, information was extracted on study design, sample size, implant type and bearing surface, presence of modular junctions, definitions of IP/ALTR, diagnostic modalities used, and key clinical or radiographic outcomes. Owing to substantial heterogeneity in definitions, imaging protocols, and outcome measures, no formal meta‐analysis was attempted. Instead, findings were organized thematically into epidemiology, pathophysiological mechanisms, risk factors, clinical presentation, diagnostic pathways, and management, with qualitative appraisal of the overall strength and consistency of the evidence.

### 2.4 Ethical Approval

This study was conducted in accordance with the ethical principles of the Declaration of Helsinki. The inclusion of clinical and intraoperative images was approved by the Institutional Review Board of West China Hospital, Sichuan University (Approval No. 2023‐950). Informed consent was obtained from the patient for the use of their anonymized images for publication and research purposes.

## 3 Standardized Terminology

The terminology describing periprosthetic soft tissue reactions has historically been heterogeneous, with terms such as aseptic lymphocyte‐dominated vasculitis‐associated lesion (ALVAL), adverse reaction to metal debris (ARMD), and ALTR often used interchangeably [6, 7, 8]. However, distinct definitions exist for these terms, as summarized in Table 1. IP refers specifically to a non‐neoplastic soft‐tissue mass visible on imaging or encountered during surgery around an arthroplasty [9, 10]. ALTR is a broader clinicopathologic entity encompassing any adverse tissue reaction, including both immune‐mediated and mechanical responses [7, 11]. ARMD denotes a metal‐ion‐specific subset of ALTR [1, 12], while ALVAL represents a specific histopathologic pattern associated with chronic hypersensitivity to implant components [6, 13].

**TABLE 1:** Standardized terminology for periprosthetic reactions.

Term	Core meaning	Diagnostic domain
Pseudotumor (IP)	Non‐neoplastic soft‐tissue mass around arthroplasty [9, 10]	Imaging/surgical
ALTR	Any adverse tissue reaction (immune/mechanical) [7, 11]	Clinicopathologic
ARMD	Metal‐ion–specific subset of ALTR [1, 12]	Metal interface pathology
ALVAL	Specific histopathologic pattern [6, 13]	Tissue microscopy

McGrory et al. defined ALTR as encompassing variable pathologic features, including osteolysis, bone, muscle, tendon, and capsular necrosis, cystic or fluid collections, soft‐tissue masses, and ALVAL [11]. While primarily local, these lesions may extend into adjacent soft tissues or muscles, causing neurological symptoms and vascular obstruction. ALVAL, sometimes incorrectly substituted for ALTR or ARMD, represents a specific histopathologic reaction pattern. In contrast, ARMD denotes a metal‐ion‐mediated subset of ALTR, most extensively described in patients with MoM THA [8].

For consistency in this review, the term “inflammatory pseudotumor (IP)” will be used when referring to the imaging/surgical finding of a non‐infectious, non‐malignant mass lesion occurring in proximity to THA, which may present as cystic, solid, or mixed tissue. Other terms will be used according to their specific definitions when discussing relevant literature.

## 4 Epidemiology

The true incidence of IP after THA remains uncertain due to substantial underreporting of asymptomatic cases [9] and variability in diagnostic criteria [10, 14, 15]. Studies show the need to differentiate imaging‐detected prevalence from symptomatic incidence and revision risks across bearing surfaces. This distinction highlights why IP is an increasingly recognized complication, particularly with advanced imaging like MARS‐MRI.

### 4.1 Imaging‐Detected Prevalence

Imaging studies, primarily using MARS‐MRI, reveal high prevalence rates of IP after THA, including substantial asymptomatic cases that vary by bearing type and follow‐up duration. MoM THA shows the highest rates at 40%–60% overall [14, 16, 17], with 43%–53% in screened cohorts such as 53% among 92 MoM cases reported by Kleeman et al. [18] and 53.7% by van der Veen et al. [14] while metal‐on‐polyethylene (MoP) ranges from 21.8%–41% [14, 19], appearing comparable in some long‐term analyses like Hjorth et al.'s 41% at 7.1 years postoperatively [19]. Ceramic‐on‐polyethylene (CoP) demonstrates 33%–34% at 16.9 years, with rates comparable to MoP (35%), suggesting polyethylene wear debris contributes across non‐MoM bearing surfaces [20]. Notably, Kwon et al. identified asymptomatic IP via MARS‐MRI in 15% of individuals and 36% of the overall cohort with dual‐taper modular stems, underscoring that most detected cases remain clinically silent [21]. These findings, however, face substantial challenges from heterogeneous IP definitions across reports and screening biases, which impede reliable prevalence estimation.

### 4.2 Symptomatic IP/ALTR Incidence

Symptomatic cases represent only a clinical subset of detected IPs, as a substantial proportion remain asymptomatic. Incavo et al. found IP in 36% of 83 screened patients presenting with elevated cobalt levels [22], while Kwon et al. identified symptomatic IP in 21% with dual‐taper modular stems [21]. This demonstrates that symptomatic presentation varies widely and does not equate to IP diagnosis, but symptoms like pain, instability, or swelling should prompt further evaluation with imaging.

### 4.3 Revision Risk for ARMD/IP

Revision rates for ARMD/IP reflect severe, progressive disease requiring surgical intervention. Palazzuolo et al. reported a 20% failure rate at 13 years post‐implantation, with ARMD accounting for 59% of revisions [23], while Holappa et al. documented survivorship decreasing from 94% at 5 years to 82.9% at 14.6 years in 253 large‐diameter MoM THAs, with 55.9% of 34 revisions attributed to ARMD [24]. Kwon et al. identified IP in 36% of their overall cohort with dual‐taper modular stems, many leading to revision [25], and Mariotti et al. reported a 23.8% complication rate after IP revision surgery, including 14.2% infection and IP recurrence [26]. These findings highlight the substantial clinical burden of progressive IP despite high imaging prevalence, necessitating bearing‐specific surveillance strategies.

## 5 Pathophysiological Mechanisms

IP formation in MoM THA has been associated with mechanical wear at bearing and modular junctions, corrosion processes, and adverse host immune responses to metal debris [8]. In non‐MoM bearing implants, IP formation has been increasingly reported and is thought to arise through distinct pathological mechanisms, potentially involving wear at dual modular junctions and other multifactorial processes, as discussed below.

### 5.1 Debris Sources

**FIGURE 2:**
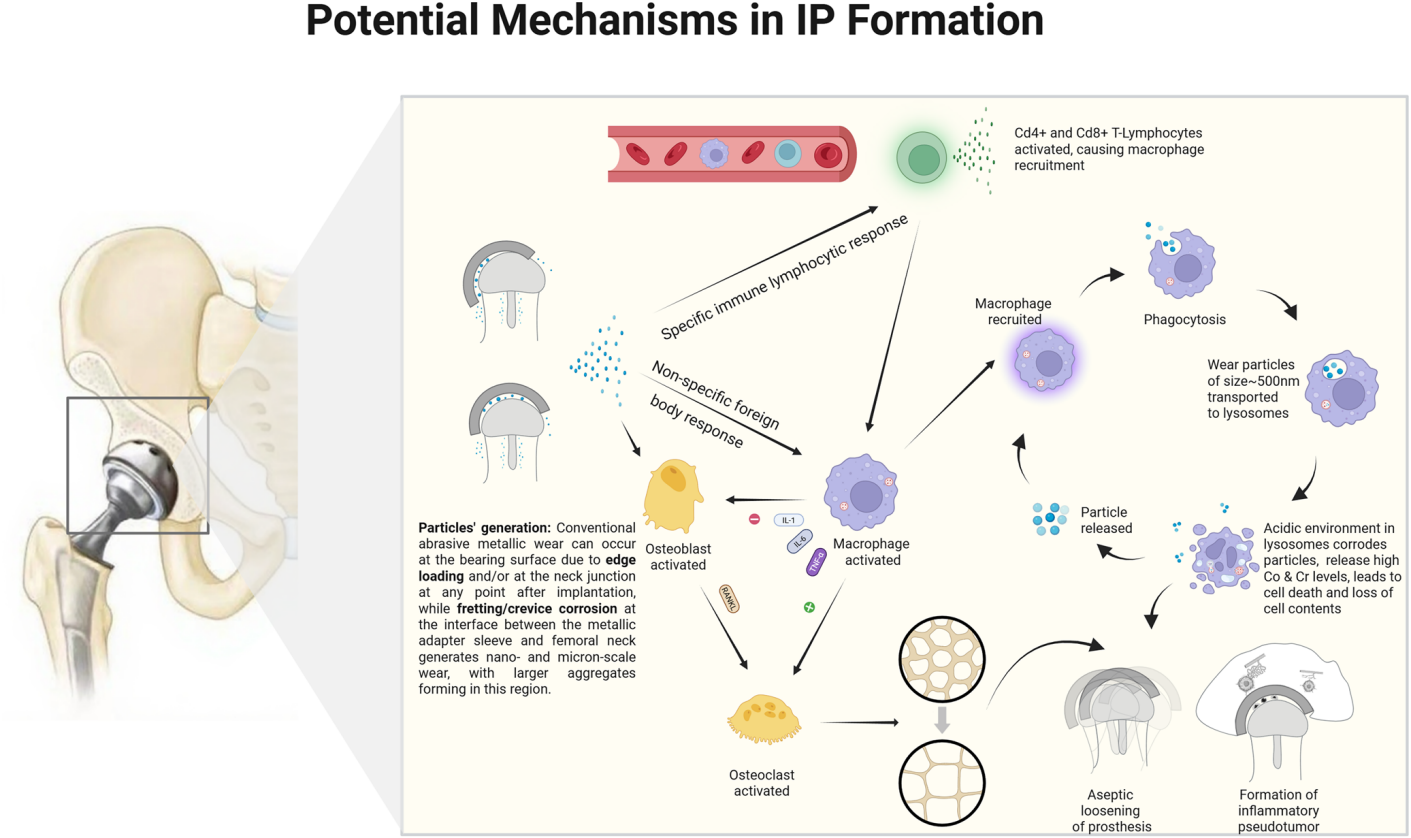
Schematic illustration of the potential mechanisms in IP formation following THA. Wear particles (metal, polyethylene, or ceramic debris) are generated at bearing surfaces or junctions within the prosthesis, including at the adapter sleeve and femoral neck. These particulates trigger either a non‐specific foreign body response, activating macrophages and osteoclasts (promoting bone resorption), or a specific immune/lymphocyte‐mediated response (including recruitment of CD4+ and CD8+ T lymphocytes). Macrophages attempt to phagocytose the particles, but ongoing particle release, lysosomal degradation, and a persistently acidic periprosthetic environment can lead to cell death and local tissue injury. These events may culminate in formation of an IP, aseptic prosthesis loosening, and periprosthetic mass. This conceptual model underscores the role of both immune‐mediated and mechanical factors in the development of IP and adverse local tissue reactions after THA. (Figure created with Biorender.com).

After MoM THA, debris generation from the bearing surfaces and modular junctions plays a central role in IP formation. Mechanical wear and corrosion at the articulating surface and at modular tapers (head–neck and neck‐stem) lead to the release of Cobalt (Co) and Chromium (Cr) particles and ions into the periprosthetic tissues. This debris elicits a local immune‐mediated inflammatory response, a form of ARMD characterized by tissue necrosis and metallosis [6, 8]. The pathogenesis of IP in dual taper modular stems involves a cascade beginning with reciprocating micromotion at the neck‐stem junction, which drives mechanically assisted crevice corrosion (fretting/tribocorrosion) [21]. This corrosion liberates metal debris, characterized by a characteristic enrichment of Co ions, reflected in elevated serum ratios (Co/Cr), a hallmark of taper corrosion as opposed to bearing surface wear [21, 25]. This debris then provokes an adverse local biological reaction, resulting in IP formation and soft‐tissue damage, which can occur even in asymptomatic patients [25]. Importantly, taper or trunnion corrosion at the head–neck and neck‐stem junctions has emerged as a bearing‐surface‐independent driver of IP, particularly in modular stems combining titanium alloy shafts with CoCr junctions. Mechanically assisted crevice corrosion at these junctions produces Co‐enriched debris and elevated Co:Cr ratios, a biochemical signature that distinguishes taper corrosion from pure bearing surface wear and has been repeatedly linked to IP and ALTR even in non‐MoM constructs. This concept of “trunnionosis” provides a unifying mechanism explaining why IPs are observed across MoM, MoP, CoP, and even CoC implants (Figure 2). Corroborating evidence from isolated case reports further broadens this etiology, demonstrating that structural failure of ceramic components, such as fracture of the femoral head in CoC bearings, can generate substantial particulate debris, which has likewise been implicated in the development of IP [27]. This mechanism is further substantiated by documented instances of ceramic head breakage in CoC implants leading to IP formation, as reported in the literature [27, 28].

The generation of wear debris from implant materials is a primary driver of periprosthetic pathology. As demonstrated by Ishida et al. excessive articular wear of a conventional polyethylene liner can produce large supra‐macroparticles, which trigger a foreign‐body giant cell reaction and the formation of symptomatic IP, distinct from metal‐induced responses [29]. This underscores the direct role of polyethylene debris in causing soft‐tissue complications. Fortunately, material science advancements have directly targeted this mechanism of failure. The systematic review by Kurtz et al. confirms that first‐generation highly crosslinked polyethylene (HXLPE) liners in hip arthroplasty reduce the mean femoral head penetration rate by approximately 70% (from 0.137 to 0.042 mm/year) and, critically, are associated with an 87% lower risk of osteolysis compared to conventional polyethylene. This profound reduction in wear debris generation fundamentally alters the biological landscape, significantly mitigating the primary pathway for both osteolytic bone loss and, by extension, the inflammatory substrate necessary for IP formation [30]. Together with taper corrosion, these data support a model in which both polyethylene wear and modular junction corrosion can independently or synergistically generate the inflammatory milieu required for IP formation in non‐MoM THA.

### 5.2 Foreign‐Body Reaction

The foreign‐body reaction to implant debris is a key driver of inflammation and IP formation. This process, described by both Hall, Pourzal, and Jacobs and Eltit et al. is characterized by macrophages that phagocytose metal particles and, in reaction, secrete pro‐inflammatory chemokines and cytokines. This resulting inflammatory cascade is a central mechanism in the development of the benign fibromas clinically identified as IPs [7, 31].

### 5.3 Immune‐Mediated Hypersensitivity

ALTRs to Co/Cr orthopedic implants most commonly manifest histologically as type IV hypersensitivity reactions, necessitating initial sensitization via metal ion exposure before subsequent immune activation through either macrophage‐dominant (CD4^+^) or lymphocyte‐dominant (CD8^+^) cell‐mediated pathways. Increased exposure to metal ions and/or biological conjugates in serum and periprosthetic tissues constitutes the principal mechanism, whereby T helper cells, functioning as antigen‐presenting cells, become sensitized, precipitating lymphocyte and macrophage activation alongside aggressive chemoattractant signaling that culminates in chronic inflammation typified by ALVALs. Specific genetic predispositions, particularly certain HLA genotypes that impair immune down‐regulation, heighten individual susceptibility, thereby potentiating high‐grade ALVAL formation and, in extreme cases, highly destructive IP development [6]. ALTRs manifesting as IP in patients with MoM THAs arise through Th‐1 cell‐mediated immune processes, whereby interface wear generates micron‐sized metal debris producing Co and Cr ions that bind to serum proteins, forming immunogenic complexes which activate naïve T‐lymphocytes and signal macrophage recruitment via antigen‐dependent mechanisms. This represents an acquired hypersensitivity response distinct from preexisting metal allergy, uncorrelated with serum ion concentrations, component wear, or malpositioning. High‐resolution HLA Class II genotyping demonstrates DQB103:01 carriage (65% in ALTR cases vs. 25% in controls; OR 5.0, 95% CI 1.1–22.5) confers elevated revision risk for symptomatic IPs, whereas DRB107:01, DQA102:01, and DQB102:01/02 alleles provide protection; combined absence of protective alleles with DQB1*03:01 yields highest risk (OR 7.1, 95% CI 1.4–36.1), independent of age, gender, or ion levels [32].

In a matched case–control study of MoM hip resurfacings, periprosthetic IPs confirmed histologically as ALVALs predominantly of intermediate‐grade group 2 (63%) or group 3 (25%) severity demonstrated significant HLA genotype associations independent of implant type (ASR/BHR matched), age, BMI, or markedly elevated pre‐revision Co/Cr ion levels (cases: mean 730/443 nmol/L vs. controls: 31/35 nmol/L; *p* < 0.002). High‐resolution next‐generation sequencing across 11 HLA loci identified DQB105:03:01 and DRB114:54:01 alleles (each 9.3% in cases vs. 0% in controls; *p* = 0.0676) as conferring elevated IP risk, while DQA103:01:01 (0% vs. 29.4%; *p* = 0.0240), DRB104:04:01 (0% vs. 23.5%; *p* = 0.0453), C01:02:01 (0% vs. 23.5%; *p* = 0.0453), and B27:05:02 (0% vs. 17.6%; *p* = 0.0855) were protective, supporting genetic modulation of metal debris reactivity through macrophage (high ion) or T‐lymphocyte hypersensitivity (low ion) pathways [33].

### 5.4 Histopathological Characterization of Periprosthetic Tissues in THA

Histopathological analysis has been essential for classifying ALTRs around THA implants, first with MoM bearings and later with corrosion at modular junctions across implant types. Early work by Davies et al. established a characteristic pattern distinct from polyethylene wear debris reactions, featuring synovial ulceration and dense perivascular lymphoid aggregates. This finding was first systematically described as suggestive of an adaptive immune response and subsequently formalized as ALVAL [13]. Further studies revealed the response is not uniform. Berstock and colleagues illustrated this spectrum by categorizing reactions into three predominant subtypes: approximately one‐third showed a superficial macrophage‐dominated response, one‐third exhibited the deep, infiltrative ALVAL‐like pattern, and one‐third demonstrated mixed features [34]. Critically, this histology correlated with clinical outcome, as revisions for the lymphocytic pattern occurred significantly earlier than for the macrophage‐dominated type, highlighting differences in biological aggressiveness [34].

This histopathological variability, coupled with the need to distinguish between failure mechanisms, necessitated standardized scoring. Two principal systems emerged: the Campbell‐ALVAL score, a 10‐point system integrating synovial integrity, inflammatory infiltrate, and tissue organization [35], and the Oxford‐ALVAL score, which focuses specifically on quantifying the perivascular lymphocytic infiltrate [36]. The Campbell score effectively differentiates tissues from high‐wear failures (lower scores, macrophage‐rich) from those revised for unexplained pain/suspected hypersensitivity (higher scores, lymphocyte‐dominant) [35]. Comparative evaluation suggests the Oxford system's targeted design may provide a more suitable tool for assessing tissues from implants revised for mechanical issues like loosening, where high‐grade lymphocytic reactions are less common [36]. Imaging grading systems for CT and MRI [37, 38] (Table 2) complement these histopathological scores by quantifying lesion severity. The consistent application of such systems is vital for correlating histological findings with clinical outcomes across studies.

**TABLE 2:** CT and MRI grading system for IP.

Classification	Description
CT grading system [39]	
Grade I	Normal or acceptable: > A 4‐ to 6‐mm thickening of the capsule
Grade II	Reactive: > Thickened capsule > 6 mm without eccentric growth relative to the capsule, with or without bulging but not beyond the prosthesis' neck
Grade III	Mild MoM disease: > Has an anterior and posterior protruding capsule
Grade IV	Moderate MoM disease: > Eccentric bulging or expansion of capsule, often seen inferomedial to prosthetic head
Grade V	Severe MoM disease: > Resembles bursitis filling bursa iliopectineal (anteriorly) or bursa subtrochanteric (posterolateral), sometimes extending into abdominal compartment
MRI grading system [38]	
Hauptfleisch methods	
Type I	Cystic mass with thin walls (wall < 3 mm)
Type II	Thick‐walled cystic mass (wall > 3 mm but less than cystic component width)
Type III	Primarily dense mass
Anderson methods	
Normal (A)	Postoperative manifestations such as hematomas, seromas
Infection (B)	High‐signal T2 wall fluid‐filled cavity with soft tissue inflammation ± bone marrow oedema
Mild MoM disease (C1)	Periprosthetic soft tissue mass or fluid‐filled cavity without hypertensive T2W signal; max diameter < 5 cm
Moderate MoM disease (C2)	C1 lesion with soft tissue mass or fluid‐filled cavity > 5 cm
Severe MoM disease	Any of the following: (1) fluid‐filled cavity through deep fascia, (2) tendon avulsion, (3) intermediate T1W soft tissue cortical or marrow signal, (4) fracture

## 6 Risk Factors for IP

IP development reflects an interplay of patient‐related, genetic, implant‐related, and surgical factors [12, 39, 40, 41] (Table 3), with heterogeneous and sometimes conflicting evidence. The following subsections summarize these domains, indicate the strength of available data, and highlight ongoing controversies.

**TABLE 3:** Summary of reported IP burden after THA by risk factor and bearing type.

Category	Risk factor	Effect	Data type	Evidence strength	References
Patient	Female sex	61%–69% symptomatic cases	Symptomatic prevalence	Moderate	[10, 40]
Patient	Metal hypersensitivity	↑ (Suspected)	Not quantified	Low	[42]
Genetic	HLA alleles (DQB105:03:01, DRB114:54:01 ↑ risk; DRB1*07:01 protective)	↑ Revision risk (OR 5.0–7.1)		Moderate	[32, 33]
Implant	MoM bearing	40%–60%	Revision association	High	[14, 16, 17]
Implant	MoP bearing	21%–41%	Imaging prevalence	High	[14, 19]
Implant	CoP bearing	33%–35%	Imaging prevalence	Moderate	[20, 29]
Implant	Large head (≥ 36–38 mm)	53.7% MoM vs. 4.5% small‐head	Imaging prevalence	High	[14, 38]
Implant	Stem modularity (dual‐taper)	15% asymptomatic, 36% overall; ↑ revision	Imaging prevalence + revision risk	Moderate/controversial	[21, 25, 43]
Surgical	Cup malposition (> 55° inclination)	↑ Edge‐loading/wear	Revision risk	Moderate	[44]

*Note:* Data Type definitions: Imaging prevalence = IP/pseudotumor detected on MARS‐MRI, CT, or ultrasound (often includes asymptomatic cases); Symptomatic prevalence = Clinical symptoms (pain, instability, swelling) attributed to IP/ALTR; Revision risk = Proportion of hips revised where IP/ARMD was primary indication, or revision rate where IP caused failure; Revision association = Genetic/clinical factors linked to higher revision rates for IP/ALTR.

### 6.1 Patient‐Related Factors

Patient‐related factors most consistently associated with IP include sex [7, 10, 40], possible differences in immune reactivity, and suspected metal hypersensitivity [42], while the roles of age and activity level remain less clearly defined. Several series report a predominance in women: Persson et al. observed that 69% of patients revised for symptomatic IP were female [40], and Cerchiaro et al. similarly found 61.9% of their IP cohort were women [10], suggesting a sex‐linked susceptibility, although causality is not established. Proposed mechanisms include hormonal influences, differential immune regulation, and increased prior exposure to metal allergens, but these hypotheses remain speculative and require further validation. Younger age and high activity levels have been suggested to increase wear and metal ion production, potentially elevating IP risk. Hjorth et al. reported that younger, physically active patients with MoM hip articulations exhibited significantly higher serum‐ion concentrations of Cr and Co, which were correlated with a higher risk of IP formation [45]. However, robust, IP‐specific data separating these effects from implant and surgical variables are limited.

Overall, patient‐related risk factors, particularly female sex and suspected metal allergy, appear important but are supported mainly by observational and retrospective studies, with limited ability to control for confounders. The strength of evidence is moderate for female sex (based on consistent symptomatic prevalence data) and low for metal hypersensitivity and activity level (based on mechanistic hypotheses and indirect associations).

### 6.2 Genetic Factors

Genetic predisposition, especially specific HLA alleles, has emerged as a substantial host factor in IP susceptibility, underscoring the role of delayed‐type hypersensitivity mechanisms. Kilb et al. reported that HLA‐DQB1*03:01 was present in 65% of patients revised for symptomatic IP after MoM THA, while alleles such as DRB1*07:01, DQA1*02:01, and DQB1*02:01/02 appeared protective; the absence of DRB1*07:01 in DQB1*03:01 carriers further increased revision risk [32]. Sheridan et al. similarly identified DQB1*05:03:01 and DRB1*14:54:01 as at‐risk alleles and DQA1*03:01:01, DRB1*04:04:01, C*01:02:01, and B*27:05:02 as protective in MoM hip resurfacing cohorts [33].

These consistent associations across studies support a meaningful immunogenetic contribution to IP pathogenesis. However, cohorts remain relatively small and often ethnically restricted. Therefore, HLA typing is not yet recommended for routine clinical risk stratification outside research settings, given the current absence of validated predictive algorithms or cost‐effectiveness data. The strength of evidence is moderate, based on clear revision risk associations in case–control studies.

### 6.3 Implant‐Related Factors

Implant‐related variables, including bearing surface, femoral head size, modularity, and alloy pairing, represent the most extensively studied risk domain, though results are heterogeneous and often interdependent.

#### 6.3.1 Bearing Surface

**FIGURE 3:**
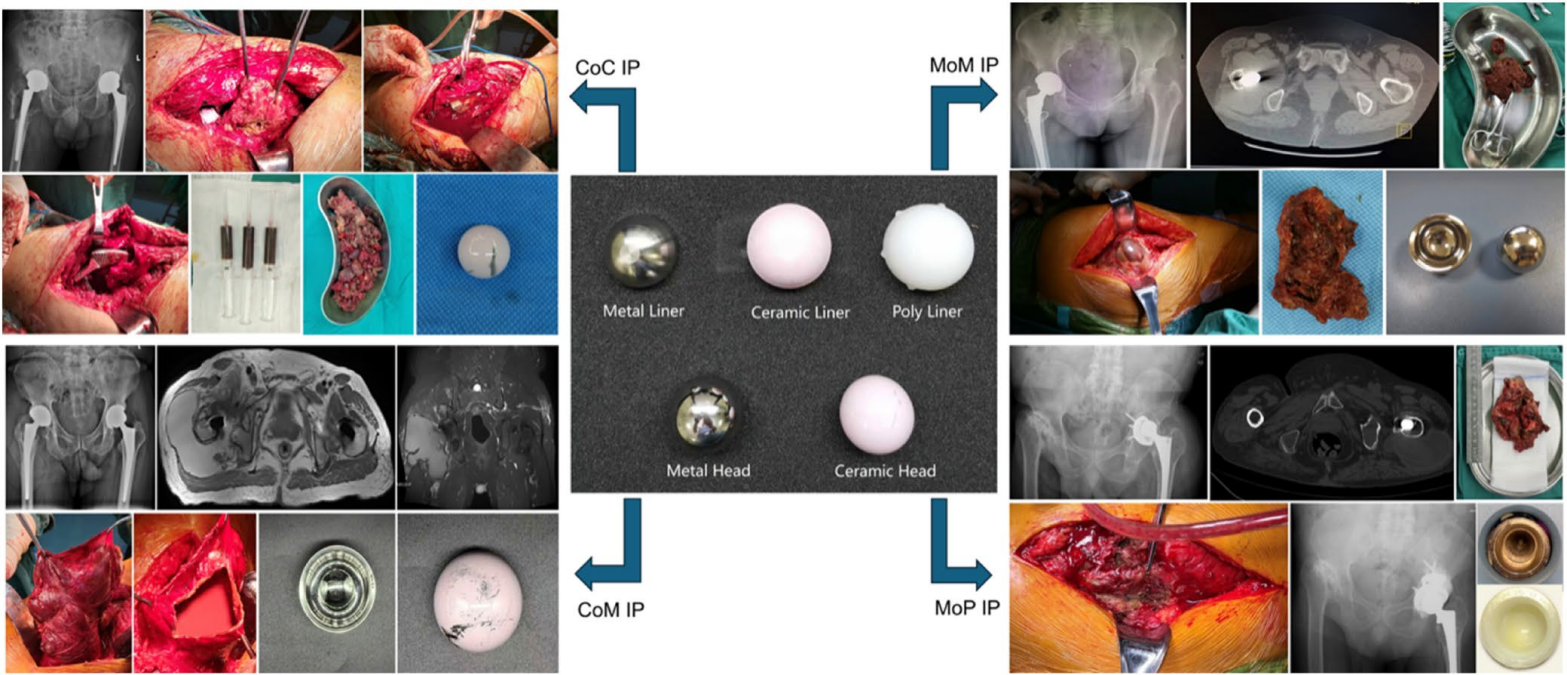
Multimodal diagnostic appearances of IPs in THA for different bearing surfaces. The central panel shows implant components (liners and heads) for metal‐on‐metal (MoM), ceramic‐on‐ceramic (CoC), ceramic‐on‐metal (CoM), and metal‐on‐polyethylene (MoP) bearings. Surrounding panels demonstrate characteristic radiographic, intraoperative, gross, and imaging features of IPs encountered with each bearing surface, including: X‐rays, CT/MRI, and intraoperative views illustrating typical IP morphologies, retrievals of debris and tissue masses associated with IPs, polyethylene wear, and metal/ceramic head and liner conditions. This composite visually underscores that IP formation can occur with all major modern bearing couples and demonstrates the spectrum of diagnostic appearances and intraoperative findings associated with each.

All bearing couples are associated with IP (Figure 3), but the MoM THA series reports the highest imaging‐detected IP prevalences (40%–60%) [14, 16, 17], higher ARMD‐related revision rates [46, 47], and declining survivorship, contributing to the global decline and recall of several MoM systems [10, 48]. Large‐head MoM THA and resurfacing series demonstrate these patterns most prominently, with Holappa et al. documenting survivorship decreasing from 94% at 5 years to 82.9% at 14.6 years [47].

Nevertheless, IP is not exclusive to MoM THA [3, 29, 49]. MoP and CoP constructs also demonstrate substantial IP incidence, with Ishida et al. reporting similar rates in CoP (33%) and MoP (35%) at 16.9 years, implicating polyethylene wear particles as a contributor in non‐MoM hips [20, 29]. The use of HXLPE liners is associated with an 87% reduction in the risk of osteolysis compared to conventional liners [30], yet IP formation remains reported even with HXLPE [19].

In CoC bearings, IP has been primarily attributed to ceramic component fracture [27, 28], leading to severe wear and multiple IP formation [27]. However, IP has also been reported in the absence of fracture, arising directly from CoC bearing surfaces [28], and some researchers suggest ARMD in these implants might instead originate from corrosion at the neck‐stem junction [3] Furthermore, most reported CoC‐associated IP cases remain poorly understood [49, 50], a diagnostic challenge compounded by infectious complications that can mimic or coexist with IP [49]. This variability underscores the complex and multifactorial nature of IP pathogenesis 1. From a contemporary perspective, the decline in MoM usage shifts attention to non‐MoM IPs, where polyethylene wear and, critically, trunnion corrosion at modular junctions account for a growing proportion of ALTRs. Reports of IP in MoP, CoP, and CoC constructs in the absence of gross component malposition or extreme bearing wear strongly implicate taper corrosion as a common final pathway for metal‐mediated tissue damage.

#### 6.3.2 Femoral Head Size

Large‐diameter femoral heads (particularly > 36–38 mm) were introduced to reduce dislocation and increase range of motion [48, 50, 51], but have been associated with increased volumetric metal wear in MoM constructs [52], potentially augmenting ARMD and IP risk. Van der Veen et al. observed low IP incidence (4.5%) in small‐head MoM THA comparable to standard MoP THA (9.4%) over > 13 years, whereas their prior study of large‐head MoM reported markedly higher IP rates (53.7% MoM; 21.8% MoP) [38], suggesting an interaction between head size, taper geometry, and wear. However, not all large‐head series demonstrate uniform failure, indicating that head size is an important but not isolated determinant, modulated by implant design, metallurgy, and surgical technique. The evidence is strong for an association with imaging prevalence, particularly in MoM hips.

#### 6.3.3 Femoral Stem Modularity and Alloy Pairing

Modular stem designs offer intraoperative flexibility and anatomical restoration but introduce additional junctions vulnerable to fretting, corrosion, and mechanically assisted crevice corrosion [43, 53, 54]. Comparative studies of MoM systems with and without modular necks have reported higher rates of ALTR in modular constructs, implicating neck‐stem corrosion independent of bearing surface [43].

Alloy pairing plays a critical role; the combination of titanium alloy (Ti6Al4V) stems with Co‐Cr modular necks increases fretting corrosion risk [12]. This has prompted recalls of several dual‐modular stems following clusters of ALTR‐related failures [43, 53]. Nonetheless, some series report no clear association between modularity and ALTR [52], and the overall evidence remains mixed and controversial, suggesting that specific design features and taper geometry may be more important than modularity per se, as trunnion flexibility influences corrosion risk across designs [55]. The association is primarily supported by revision risk data, including documented taper junction mismatches, modular dual‐mobility utilization trends (6.7% → 12% from 2012–2018), and specific implant recalls [53, 54, 56]. These observations underscore that trunnion corrosion is not confined to MoM bearings, but can drive IP formation in any construct that combines susceptible taper design, alloy pairing, and loading conditions, making it a central pathophysiologic mechanism in modern non‐MoM THA.

### 6.4 Surgical and Positional Factors

Surgical technique and component positioning influence local biomechanics, wear patterns, and the risk of edge‐loading, and thereby may modulate IP risk, although high‐quality quantitative data are limited. While the risk of edge‐loading from adverse cup positioning was historically implicated in IP formation due to increased wear debris, the study by Matthies et al. [44] found that IPs were equally common in well‐positioned hips, suggesting edge‐loading is not a primary or necessary causative factor. Although many clinical series acknowledge the importance of component position and technique, most are retrospective and not powered to isolate surgical variables from implant‐ and patient‐related factors, and formal thresholds for high‐risk orientations remain debated. Consequently, surgical and positional factors should be regarded as modifiable contributors that may amplify or mitigate IP risk in the context of a given implant construct and host profile, rather than isolated primary drivers.

## 7 Clinical Manifestation

The clinical presentation of IP is highly variable, ranging from entirely asymptomatic to severely symptomatic cases [39]. Pain, often localized to the groin, thigh, or buttock, is the most common symptom [39, 57]. Additional symptoms include swelling, palpable masses, nerve palsy, venous compression mimicking deep vein thrombosis, and even mechanical symptoms like subluxation or dislocation due to the mass effect of IP [16, 58, 59]. Prosthesis dislocation may be precipitated by the physical impingement of the IP on the prosthetic components, leading to progressive displacement seen on imaging. Given this variability, clinical suspicion should remain high in patients presenting with unexplained pain, swelling, or mechanical symptoms after arthroplasty. Careful correlation with imaging and prior surgical history is essential to confirm the diagnosis, assess the extent of the lesion, and guide appropriate management planning.

## 8 Diagnostic Modalities

The diagnosis of IP is challenging due to its variable clinical presentation and the need to differentiate it from other causes of arthroplasty failure, most critically periprosthetic joint infection (PJI). Consequently, a systematic, multimodal diagnostic approach is essential. No single test is definitive; instead, diagnosis relies on the correlation of clinical findings, laboratory investigations, and advanced imaging. The following sections detail the role of each modality and integrate them into a stepwise, evidence‐based diagnostic algorithm designed to optimize accuracy and guide timely management.

### 8.1 A Stepwise Diagnostic Algorithm for IP

Given the variable and non‐specific clinical presentation of IP, a systematic, evidence‐based diagnostic pathway is essential to distinguish it from other causes of arthroplasty failure, most critically PJI. The following stepwise algorithm integrates clinical assessment, laboratory analysis, and advanced imaging to optimize diagnostic accuracy and guide timely intervention.

### 8.2 Initial Clinical and Serological Evaluation

The assessment of any painful hip after THA must commence with a detailed history and physical examination. For instance, in a study of painful IPs following MoM THA, the evaluation meticulously documented pain characteristics and location, which were key factors in diagnosis [57]. When IP is suspected based on this initial evaluation, often indicated by groin pain, a palpable mass, or a history of MoM bearing surfaces, advanced imaging and biopsy become crucial, as highlighted in the management studies of this condition [10].

A thorough implant history is paramount, noting MoM bearings or modular components associated with higher ALTR risk [10, 12]. The critical need to exclude PJI during the evaluation of a painful MoM arthroplasty is underscored by standard pre‐operative diagnostic protocols. As demonstrated in a surgical case series of revisions for ALTRs, a fundamental component of the pre‐revision workup is laboratory screening, including the collection of C‐reactive protein (CRP) blood levels, to rule out septic etiology before attributing symptoms to IP [26]. This practice is further illustrated in clinical case reports, where concurrent screening with both erythrocyte sedimentation rate (ESR) and CRP is described as a part of the systematic evaluation to rule out PJI, a primary differential that can mimic or coexist with an ALTR [16]. Together, this evidence highlights the established role of inflammatory markers, with CRP as an essential initial test, in the differential diagnosis between ALTR and infection.

### 8.3 Radiographic Assessment and Systemic Metal Ion Analysis

Standard anteroposterior pelvis and lateral hip radiographs form the foundational imaging evaluation. Their principal value lies in assessing component position, fixation integrity, and the presence of osteolysis or gross mechanical failure [60]. However, a critical limitation is their inability to visualize soft‐tissue pathology directly; consequently, radiographs may appear entirely normal. As shown in Figure 3, plain radiography may miss IP, whereas ultrasound, CT, and MRI provide complementary, high‐contrast visualization of the lesion.

In patients with MoM articulations or modular junctions, subsequent measurement of whole‐Co and Cr ion levels is critical. A threshold > 7 μg/L (ppb) is widely considered a level of concern with high specificity for ALTR [60, 61]. This imperfect correlation is underscored by the study of Incavo et al. who detected IP via MRI in 36% of a screened cohort with elevated Co levels, noting that while all patients with IP had serum Co > 8 ppb, low serum levels did not definitively exclude the diagnosis [22].

The clinical management of MoM‐THA patients has historically relied on systematic surveillance using serum Co and Cr ion levels, with a threshold of > 7 ppb widely employed as a level of concern for ALTR [61]. This paradigm is exemplified in the study by Pogliacomi et al. which stratified 383 MoM‐THA patients based on this threshold. Their findings reinforce its practical utility, demonstrating a distinct cohort with elevated wear (> 7 ppb) that warranted advanced imaging. Crucially, however, this study also underscores the fundamental limitation of systemic ion levels: patients with concerning ion levels (7–60 ppb) reported clinical scores identical to those with safe levels, revealing a significant disconnect between biochemical evidence of wear and patient symptoms [60]. This observed insensitivity and delay in systemic biomarker elevation provide the clinical rationale for the evolving diagnostic landscape. Recent evidence suggests that lower blood ion thresholds may improve predictive accuracy for ALTR, and more importantly, that direct measurement of metal ions in synovial fluid offers a markedly more sensitive and specific biomarker, reflecting the local joint pathology more accurately than diluted systemic levels [61]. Thus, while studies like Pogliacomi et al. validate the use of the > 7 ppb threshold in existing algorithms, their data simultaneously highlight its diagnostic shortcomings and support the investigation into more precise, localized biomarkers for earlier and more accurate detection of ALTR.

Clinicians must be aware of the imperfect correlation; a significant proportion of patients with confirmed IP present with borderline or low systemic ion levels, underscoring that normal blood ions do not rule out ALTR [22].

### 8.4 Synovial Fluid Analysis

When investigating a painful hip arthroplasty for possible ALTR, synovial fluid aspiration provides a highly accurate diagnostic tool. Specifically, a synovial fluid Co threshold of 19.75 ppb predicts ALTR with 92.3% sensitivity and 96.3% specificity, offering superior diagnostic accuracy to blood ion analysis alone [61].

In at‐risk hips, concurrent analysis of synovial fluid for Co and Cr ions provides a highly accurate diagnostic adjunct. Synovial fluid metal ion concentrations are dramatically elevated in ALTR, with synovial Co approximately 120‐fold higher than in blood, and synovial Cr over 400‐fold higher, making it a more accurate predictor of ALTR than systemic blood levels [61].

### 8.5 Cross‐Sectional Imaging: Characterization and Surgical Planning

**FIGURE 4:**
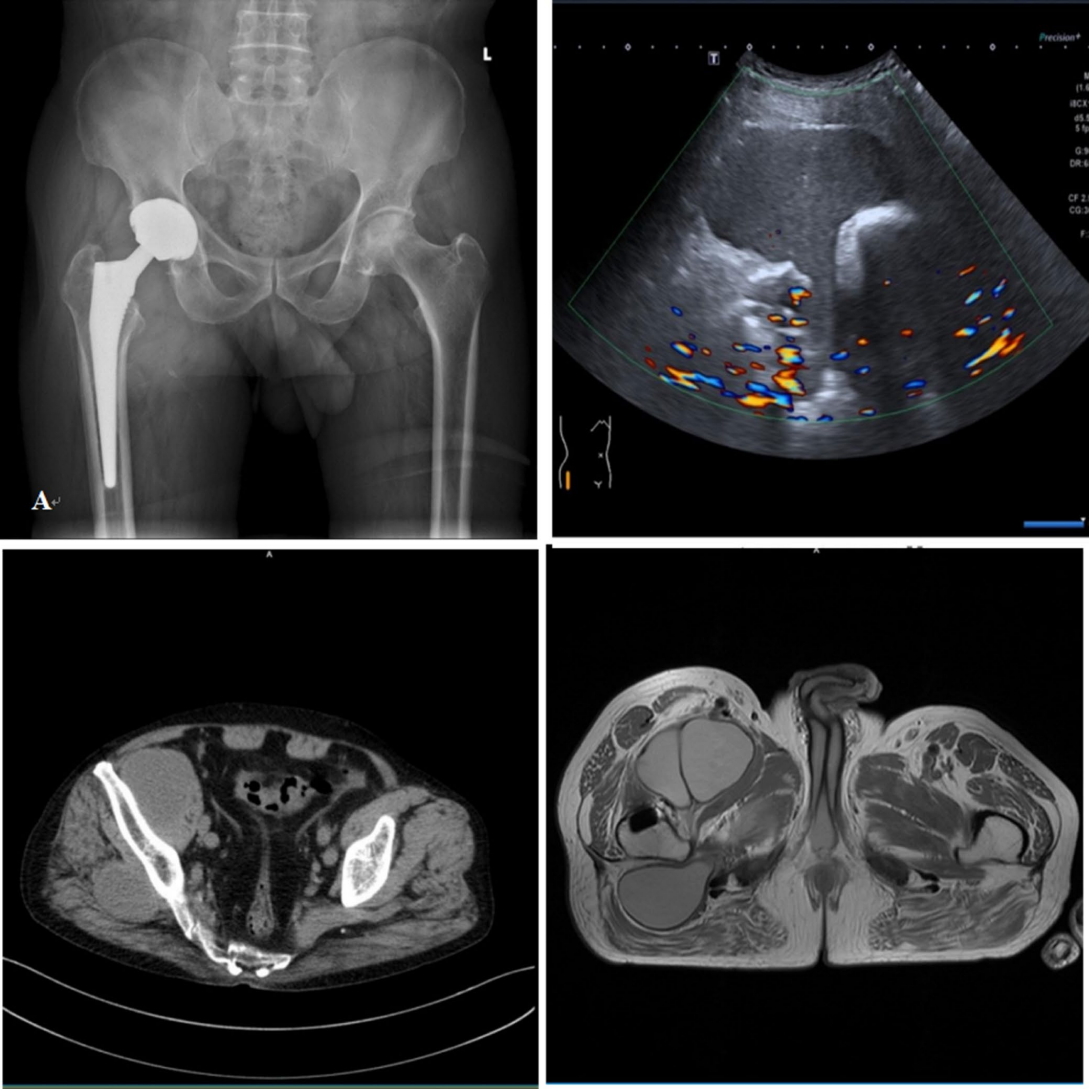
Imaging assessment of IP in a single patient demonstrating the complementary diagnostic value of each modality. The lesion is distinctly visualized on ultrasound, CT, and MRI sequences, whereas the pelvic radiograph obtained in the radiologic department fails to demonstrate the lesion conspicuously. This disparity highlights the limited sensitivity of plain radiography for detecting IP relative to ultrasound, CT, and MRI. (A) Plain pelvic radiograph. (B) Ultrasound with Doppler. (C) Axial CT. (D) Axial MRI.

Cross‐sectional imaging is required to confirm the presence of an IP, define its morphology (cystic, solid, or mixed), and plan revision surgery. A multimodal, complementary approach is recommended, with each modality offering distinct strengths (Figure 4) [60, 62, 63].

Ultrasound offers a cost‐effective, accessible diagnostic tool with high accuracy [12, 62, 64, 65]. Choe et al. developed a standardized ultrasonographic protocol targeting periprosthetic muscles in hip resurfacing arthroplasty patients. In 45 hips followed for a mean of 8.6 years, ultrasound matched MRI accuracy in identifying abnormalities including IP, infection, and bursitis [65], affirming its reliability and feasibility, especially for patients contraindicated for MRI (e.g., claustrophobia) [60]. Limitations include operator dependency and reduced reliability for evaluating deep structures in obese patients [60, 65].

Computed Tomography (CT) provides superior evaluation of bone stock, component version, and the extent of osteolysis, which is critical for preoperative planning [63, 65, 66]. Historically, its utility was limited by metal artifact and poor soft‐tissue contrast for ALTR characterization. However, advancements, including iterative reconstruction and dedicated metal artifact reduction algorithms, have significantly enhanced its diagnostic utility, allowing CT to effectively complement MARS‐MRI in the comprehensive management of these patients [66].

MARS‐MRI remains the gold standard for visualizing soft tissue involvement in IP, including solid and cystic masses, muscle atrophy, and tendon avulsions [4, 63, 67]. It provides unparalleled visualization of IP characteristics, muscle integrity (e.g., abductor destruction), and the extent of soft‐tissue damage. MARS‐MRI is indicated for symptomatic patients or those with elevated blood metal ions and is central to surveillance, having identified IPs in 36% of patients with a modular stem, including 15% of asymptomatic individuals [22]. Smeekes et al. evaluated 240 MoM THA patients and observed a 59% IP prevalence in high‐risk groups versus 43% in controls and none in low‐risk patients. The study found moderate interobserver agreement across MRI grading systems, with the Matthies score performing best, affirming MARS‐MRI's clinical utility despite grading variability [37].

### 8.6 The Evolving Role of Advanced Metabolic Imaging

The application of 18F‐FDG‐PET/MRI in the evaluation of periprosthetic abnormalities is currently not part of routine diagnostic algorithms. Existing evidence, primarily from preliminary and small‐scale studies, positions it as a specialized adjunct. For instance, Kimura et al. in a preliminary report of 11 patients, demonstrated that PET/MRI could identify fluorodeoxyglucose uptake within metal artifact zones corresponding to sites of tissue reaction. However, they concluded it was useful only as an auxiliary diagnostic tool and explicitly noted that MARS‐MRI is preferable for clinical practice due to practical and safety considerations [4]. Significant barriers to routine adoption include the lack of established diagnostic criteria, variability in quantitative uptake values, absence of data in asymptomatic populations, and the modality's cost and radiation burden. Therefore, its use is best reserved for complex, diagnostically challenging scenarios within a research or highly specialized clinical context [4].

### 8.7 Integrated Diagnostic Pathway

**FIGURE 5:**
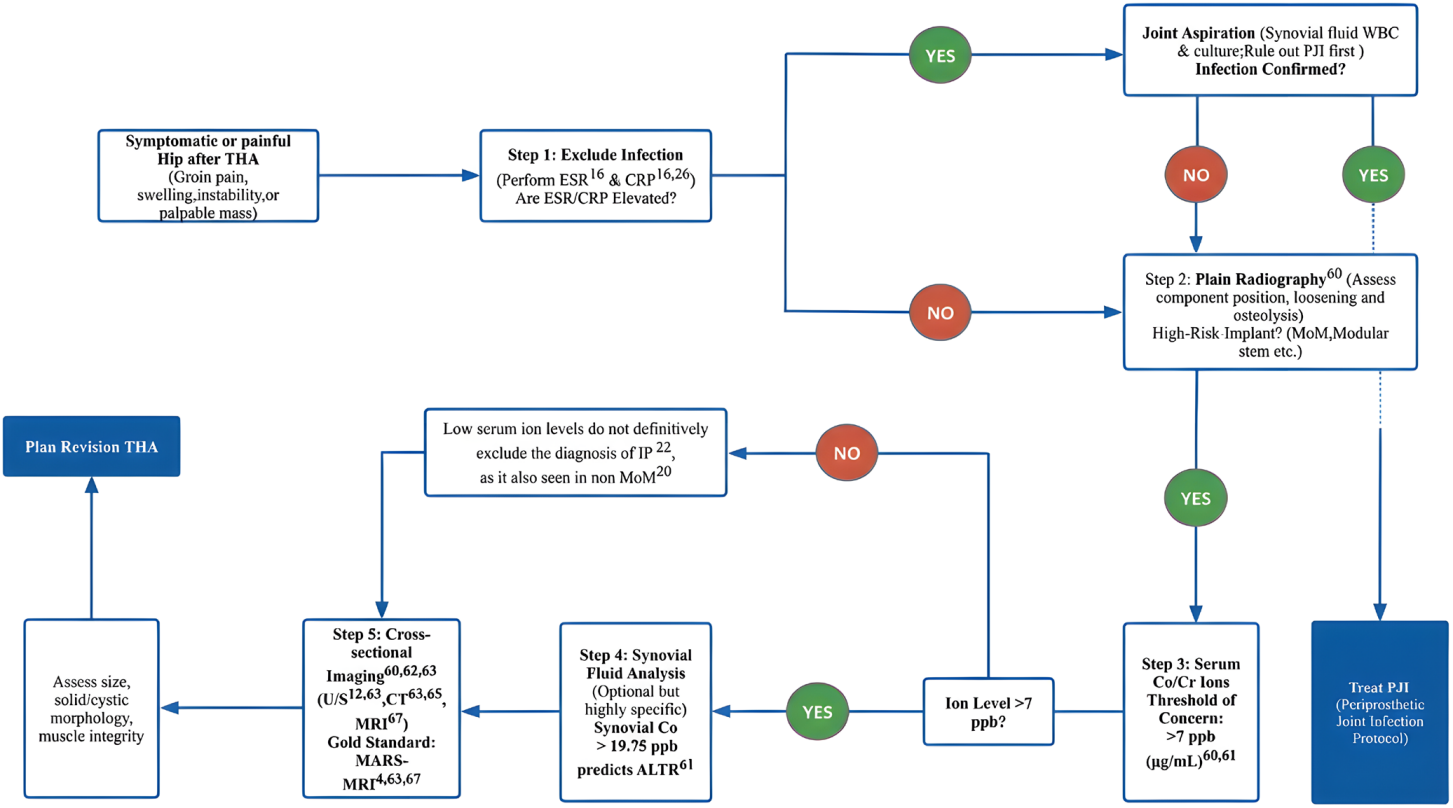
This evidence‐based pathway integrates clinical assessment, laboratory testing, and advanced imaging to distinguish IP from periprosthetic joint infection (PJI) and other causes of THA failure. Key decision points incorporate serum cobalt threshold (> 7 ppb), synovial fluid analysis, and MARS‐MRI for soft tissue characterization. ALTR, adverse local tissue reaction; CRP, C‐reactive protein; ESR, erythrocyte sedimentation rate; MoM, metal‐on‐metal.

The proposed diagnostic algorithm (Figure 5) emphasizes a logical sequence: initial exclusion of infection, risk stratification via radiographs and systemic metal ions, definitive adjunctive testing via synovial fluid analysis when indicated, and culminating in advanced cross‐sectional imaging, primarily MARS‐MRI for definitive characterization and surgical planning. This structured, evidence‐based approach ensures timely and accurate diagnosis, which is critical for intervention before the onset of irreversible soft‐tissue damage such as abductor destruction, thereby improving patient outcomes.

## 9 Management

Treatment of IP aims to achieve a painless, stable hip joint while preserving functional range of motion; however, outcomes are frequently influenced by the extent of muscle damage caused by IP. Management is tailored to individual patients, considering symptom severity, lesion size, implant stability, and patient‐specific factors, with options ranging from conservative monitoring to complex revision surgery [26, 68]. Although an optimal surgical approach remains undefined, revision is generally advised before the onset of irreversible abductor muscle damage. Several experts recommend annual MARS‐MRI surveillance for MoM bearings THA beginning 1–2 years postoperatively to identify asymptomatic IP early and enable timely surgical intervention [22].

### 9.1 Non‐Operative

A review of prevailing management principles, as summarized by Cottino et al. delineates specific criteria for non‐operative surveillance. They indicate that for patients with an established IP, the absence of pain, functional impairment, and elevated metal ion levels favors a course of ongoing observation over revision surgery [69]. This clinical rationale focusing on symptoms, function, and serology is widely acknowledged. Supporting this view, a separate surgical study also explicitly references that asymptomatic IPs without elevated ions are candidates for watchful waiting or continuous surveillance, noting that their own analysis was limited to patients who underwent operative intervention [10].

This clinical rationale focusing on symptoms, function, and serology is conceptually reflected in the novel PCS (Pseudotumor; Cup status; Stem status) classification system proposed by Stimolo et al. Their system operationalizes these factors, stating that non‐operative management of IPs should be considered when the implants are stable with minor bone loss (designated C1 S1) and the IP is asymptomatic with extra pelvic location and normal metal ion levels (designated Pae). However, this is a new classification and a proposed treatment algorithm [9]. The authors' study retrospectively analyzed only patients who underwent surgical revision. Therefore, while its criteria align with established clinical logic, the specific recommendation for non‐operative management based on the PCS classification remains a theoretical proposal from the authors; its application and outcomes for a conservatively managed cohort were not tested or reported in their study.

### 9.2 Operative Treatment

Numerous operative techniques are available for the treatment of IP. This review focuses on the commonly employed current treatment modalities. The choice of treatment largely depends on the patient's physical condition, IP size, its proximity to neurovascular structures, presence or absence of active infection, bone stock quality, and the expertise of the surgical team.

The decision to proceed with revision arthroplasty for symptomatic IPs is guided by a constellation of clinical and radiographic factors. While the presence of debilitating symptoms such as pain, swelling, and functional impairment forms the primary indication, contemporary management further stratifies surgical urgency and approach based on lesion characteristics [10]. As elucidated by Cerchiaro et al. beyond symptomatology, key drivers for intervention include IP size and progression, with the authors proposing a diameter of ≥ 7 cm as a significant benchmark and the extent of periprosthetic osteolysis, particularly major combined acetabular and femoral defects classified as Paprosky type III or IV. Although elevated serum Co and Cr levels are acknowledged, Cerchiaro and colleagues emphasize that clinical and radiographic findings should carry greater weight than metal ion concentrations alone, which can be variable. Neurovascular compromise, while less common, remains a well‐recognized absolute indication referenced in the broader literature [10]. Evidence indicates that 3D printing technology can facilitate planning and execution of complex revisions in cases of substantial bone loss, with patient‐specific 3D‐printed models aiding surgical strategy [10, 17].

### 9.3 One‐Stage Versus Two‐Stage Revision for IPs

The optimal surgical strategy for IPs complicating MoM THA remains debated, with one‐stage complete excision and revision versus staged approaches differing primarily in their handling of infection risk and soft‐tissue compromise. Mariotti et al. reported favorable 3‐year outcomes using a one‐stage approach complete IP resection followed by revision to non‐MoM bearings (predominantly ultra porous cups with ceramic liners) in 85.7% of 21 consecutive cases, yielding significant Harris Hip Score improvements (50.3 to 88.3), normalization of serum metal ions, and no re‐revisions despite a 23.8% complication rate (infections, dislocation, psoas impingement) and 14.2% recurrence. However, 14.2% required upfront resection arthroplasty due to intraoperative sepsis, underscoring the challenge of pre‐operative infection detection amid metallosis‐altered inflammatory markers (e.g., unreliable CRP, synovial aspiration, and frozen sections [26]). Practical criteria to guide decision‐making emphasize a high index of suspicion for infection, given reported rates of 14%–30% in MoM revisions, often occult within ALVAL‐damaged tissues. One‐stage revision is suitable when pre‐operative workup is reassuring (normal/mildly elevated CRP/ESR, negative aspiration), intraoperative findings show no purulence or concordant cultures, and local conditions permit radical excision plus stable reconstruction (Paprosky I‐II defects, viable capsule/abductors). Conversely, a resection‐first or two‐stage protocol is indicated for moderate to high infection suspicion (sinus tract, purulent fluid, positive cultures), extensive necrosis precluding complete debridement, or massive bone/soft‐tissue loss rendering immediate fixation unreliable. This tailored approach minimizes re‐revision risk while acknowledging that incomplete excision elevates recurrence and that non‐MoM couplings with optimized position are critical to halt debris generation [26].

Some scholars argue that IP location and the resulting mass effects on adjacent structures may necessitate multidisciplinary management and, in certain cases, staged surgical intervention [70]. Radical procedures such as hip disarticulation should be regarded as exceptional salvage options, reserved for cases in which limb preservation is not feasible.

Based on the provided clinical update by Matharu et al. the literature indicates that in revision arthroplasty for ARMD, the recommended bearing is a ceramic femoral head combined with an HXLPE liner. This is advised because this combination minimizes the risk of generating further metal wear debris and corrosion, thereby reducing the potential for ARMD recurrence [71], a principle demonstrated in clinical practice by cases such as that of Naik et al. [72], where revision of a failed metal‐bearing articulation to ceramic‐on‐HXLPE successfully lowered elevated serum metal ion levels and resolved the IP. In contrast, MoM bearings are not recommended and their re‐implantation is universally accepted to be avoided, as they represent a continued source of high wear and are associated with poor revision outcomes; this is underscored by the rapid onset of destructive IP and cup loosening seen with Ceramic on Metal (CoM) bearings, which share this risk. Furthermore, the evidence cautions against performing isolated modular component exchanges (head and liner only) in MoM THA, as this strategy is linked to a significantly higher risk of re‐revision compared to revising all components, a finding supported by the common intraoperative discovery of extensive osteolysis and component loosening that necessitates full acetabular reconstruction, as was required in the reported case. For cases with instability, options such as large‐diameter ceramic heads, dual‐mobility cups, or constrained liners may be considered, although no single option is deemed superior [71].

## 10 Future Research Directions and Unresolved Questions

Despite expanding knowledge on IP after THA, several key questions remain unanswered. First, the precise triggers of IP in non‐MoM constructs remain poorly defined, particularly the relative contributions of polyethylene wear versus taper corrosion at modern trunnions across different stem and head designs. Large, multicenter registry‐imaging linkage studies with standardized definitions of IP and ALTR are needed to quantify bearing‐independent risks and to refine surveillance thresholds. Second, the role of host genetics and immunogenetics warrants further investigation. Current HLA associations derive from relatively small, ethnically homogeneous cohorts and are not yet suitable for clinical risk stratification; future studies should integrate high‐throughput genotyping with detailed phenotyping of ALVAL histology, systemic and synovial metal ion levels, and longitudinal outcomes to develop and validate predictive models. Third, there is a need to optimize diagnostic algorithms in the era of declining MoM use. Research should clarify how best to combine blood and synovial metal ion thresholds with ultrasound, CT, and MARS‐MRI in non‐MoM hips, including cost‐effectiveness analyses and the role of emerging modalities such as PET‐MRI in complex or equivocal cases. Fourth, evidence‐based surveillance and treatment thresholds for asymptomatic IPs remain uncertain. Prospective cohorts with protocolized imaging and predefined criteria for revision are required to determine when observation is safe and when early intervention prevents irreversible soft‐tissue damage without exposing patients to unnecessary surgery.

Finally, future research should evaluate implant design and material innovations, including next‐generation tapers, alternative alloy pairings, and advanced polyethylene formulations, in terms of their long‐term impact on ALTR and IP incidence. Collaborative registries that systematically capture metal ion data, imaging, and histology will be pivotal in addressing these unresolved issues.

## 11 Conclusion

While historically linked to MoM bearings, current evidence demonstrates that IP is increasingly encountered in non‐MoM THA, driven by polyethylene wear and, above all, trunnion corrosion at modular junctions, which now represents a core pathological mechanism across bearing surfaces. The current consensus identifies IP as primarily a delayed‐type hypersensitivity reaction, with incidence and presentation influenced by sex, genetic predisposition, and exposure to wear or corrosion debris. Particulate debris modulates macrophage responses, contributing to the pathophysiology. While the precise etiology remains uncertain, accumulating evidence supports a multifactorial origin. Numerous diagnostic modalities have demonstrated utility in detecting IP, with ultrasound representing a cost‐effective, accessible option, especially in resource‐limited settings. Although several treatment strategies yield favorable outcomes, complex cases may require intervention at specialized centers with multidisciplinary expertise.

## Author Contributions

**Steve T. L. Pambayi:** data curation, writing – original draft, writing – review and editing. **Zhi Zhao:** writing – original draft, formal analysis, investigation. **Hongjun Peng:** data curation, investigation. **Ruihao Xia:** investigation, visualization. **Xishan Zhu:** data curation, formal analysis. **Wangdui Suolang:** formal analysis, investigation. **Gang Wang:** investigation, data curation. **Xiaoou Li:** visualization, writing – review and editing. **Yi Zeng:** conceptualization, supervision, resources, writing – review and editing. All authors have read and approved the final submitted manuscript.

## Funding

This work was supported by the 2023 National Key Research and Development Program of China (Grant No. 2023YFB4606700) and the Clinical Research Incubation Program of West China Hospital (Grant No. 2024HXFH008).

## Disclosure

All authors confirm that they meet the authorship criteria as defined by the latest guidelines of the International Committee of Medical Journal Editors (ICMJE). Each author has made substantial contributions to the conception, design, data collection, analysis, or interpretation of the work; has participated in drafting or critically revising the manuscript for important intellectual content; and has approved the final version for submission. All authors agree to be accountable for all aspects of the work and are in full agreement with the content of the manuscript and its submission to this journal. No automation tools were used for study selection; human reviewers performed all exclusions.

## Conflicts of Interest

The authors declare no conflicts of interest.

## Data Availability

The data that support the findings of this study are available from the corresponding author upon reasonable request.
